# Application of analyzer based X-ray imaging technique for detection of ultrasound induced cavitation bubbles from a physical therapy unit

**DOI:** 10.1186/s12938-015-0085-6

**Published:** 2015-10-19

**Authors:** Zahra Izadifar, George Belev, Paul Babyn, Dean Chapman

**Affiliations:** Division of Biomedical Engineering, College of Engineering, University of Saskatchewan, 57 Campus Drive, Saskatoon, SK S7N 5A9 Canada; Biomedical Imaging and Therapy Beamlines, Canadian Light Source Inc., University of Saskatchewan, 44 Innovation, Boulevard, Saskatoon, SK S7N 2V3 Canada; Department of Medical Imaging, Royal University Hospital, University of Saskatchewan and Saskatoon Health Region, 103 Hospital Drive, Saskatoon, SK S7N0W8 Canada; Anatomy and Cell Biology, University of Saskatchewan, 3B34 Health Sciences Building, 107 Wiggins Road, Saskatoon, SK S7N 5E5 Canada

**Keywords:** Synchrotron, X-ray imaging, Analyzer based imaging, Detection, Ultrasound, Cavitation bubble, Therapeutic ultrasound

## Abstract

**Background:**

The observation of ultrasound generated cavitation bubbles deep in tissue is very difficult. The development of an imaging method capable of investigating cavitation bubbles in tissue would improve the efficiency and application of ultrasound in the clinic. Among the previous imaging modalities capable of detecting cavitation bubbles in vivo, the acoustic detection technique has the positive aspect of in vivo application. However the size of the initial cavitation bubble and the amplitude of the ultrasound that produced the cavitation bubbles, affect the timing and amplitude of the cavitation bubbles’ emissions.

**Methods:**

The spatial distribution of cavitation bubbles, driven by 0.8835 MHz therapeutic ultrasound system at output power of 14 Watt, was studied in water using a synchrotron X-ray imaging technique, Analyzer Based Imaging (ABI). The cavitation bubble distribution was investigated by repeated application of the ultrasound and imaging the water tank. The spatial frequency of the cavitation bubble pattern was evaluated by Fourier analysis. Acoustic cavitation was imaged at four different locations through the acoustic beam in water at a fixed power level. The pattern of cavitation bubbles in water was detected by synchrotron X-ray ABI.

**Results:**

The spatial distribution of cavitation bubbles driven by the therapeutic ultrasound system was observed using ABI X-ray imaging technique. It was observed that the cavitation bubbles appeared in a periodic pattern. The calculated distance between intervals revealed that the distance of frequent cavitation lines (intervals) is one-half of the acoustic wave length consistent with standing waves.

**Conclusion:**

This set of experiments demonstrates the utility of synchrotron ABI for visualizing cavitation bubbles formed in water by clinical ultrasound systems working at high frequency and output powers as low as a therapeutic system.

## Background

One of the main interaction mechanisms that occur during the propagation of an ultrasonic wave through tissues is the possibility of acoustic cavitation. Cavitation is a complex phenomenon that involves creation, oscillation, growth and collapse of bubbles within a liquid medium to local pressure variation. A consequence of the cavitation process is the release of an enormous amount of energy in the form of an acoustic shock wave, temperature, pressure, and as visible light. When the acoustic cavitation bubble collapses close to or on a solid surface, it can collapse asymmetrically and produce high-speed jets of liquid being driving into the surface of the solid have been observed at speeds close to 400 km/h [1]. This can seriously damage the impact zone and create a newly exposed surface. This fact makes cavitation one of the important mechanism in shock wave lithotripsy for kidney stone destruction. Cavitation can also injure tissue during lithotripsy [2]. Studies [3–5] have provided indirect evidence that tissue injury response during shock-wave lithotripsy corresponds to cavitation. A number of studies have been attempted to control cavitation to obtain accelerated fragmentation while minimizing cell lysis and tissue injury [2]. Furthermore, cloud cavitation (bubble cloud) which is produced during lithotripsy is potentially the most destructive form of cavitation [2]. It has been shown that cloud cavitation is more destructive to high-speed turbo-pumps and ship propellers than the individual bubbles collapse [2]. The effect of cavitation on tissue has made a potential non-invasive therapy application of ultrasound for tissue fractionation and treatment of benign disease and cancer [6, 7]. It has been estimated that rapid adiabatic compression of gases and vapours within the bubbles or cavities produces hot spots with extremely high temperature and pressure approaching 5000 °C and 1000 atm during this collapse. As ultrasound propagates through tissue, part of its energy is absorbed by tissue which is converted to heat and energetic microbubbles that can result in cellular destruction. Damage to red blood cells [8], lung damage in mice by pulsed ultrasound in the diagnosis imaging range [9], lung lesions of pig, mice, rabbits, rats, monkey and dogs during ultrasound [3, 10–17] bring concern in ultrasonography. Furthermore, high intensity focused ultrasound treatment (HIFU) in which the ultrasound is focused into a small focal zone can damage tissue as a result of the very high temperature inside the bubbles produced, the collapse that creates a shock wave and jets, and also time duration of tissue exposure. The high temperature produced at the focal point HIFU leads to instantaneous cell death and coagulative necrosis at the focal point and with the margin of six to ten cells between live and dead cells at the edge [18]. Since the onset of cavitation and the resulting tissue damage is not predictable, high acoustic intensity is generally avoided in clinic, however cavitation is under investigation to be used as a means to enhance HIFU ablation. Another application of cavitation is in a relatively new field of medical therapy [19, 20] in which cavitation in HIFU is used for drug delivery in selectively permeable regions of tissue [21, 22]. Direct evidence of cavitation bubbles within the tissue is crucial for further development and refinement of such applications. In HIFU, gas generation, caused by cavitation, abruptly changes the pattern of heat transfer induced by ultrasound, which results in the extension of lesion from targeted area to surrounding healthy tissues [23]. The lack of cavitation bubble field probes is one of the reasons that limit the clinical development of cavitation. Consequently, it is essential to conduct a fundamental study for cavitation detection and cloud cavitation control to improve the safety and application of ultrasound therapy (such as lithotripsy and HIFU) and possibly for ultrasonography which is pervasively used for neonatal imaging.

The variation in bubble characteristics inside the cavitation field is one of the causes that make the study of cavitation characteristics so complex [24]. Once the cavitation bubbles are generated, they may undergo nonlinear oscillations during many cycles of the acoustic wave, called “stable cavitation”, or they may grow and collapse more or less violently, called “inertial cavitation” [24]. The cavitation state induced in liquid is seldom studied in most experiments. It is important to develop monitoring methods to correlate the cavitation state induced in a liquid to the biological effects observed. Since visualization of cavitation bubble field has been quite complex, workers have used some other indirect observations of macroscopic criteria to describe the induced cavitation state in a liquid [24]. However, the easiest way to study cavitation is the direct observation of the bubble field. In addition, to study the cavitation field in the body, a technique that enables detection of cavitation bubbles in tissue is required.

So far there have been a number of techniques for direct observation of the bubble field such as high speed photography [25–28], laser scattering of single bubbles, and acoustic detection of bubbles [29]. The high speed photography techniques are only applicable in in vitro systems and it is virtually impossible to capture all the bubbles considering the range of temporal and spatial scales. This technique has a very limited depth of field due to the camera [30], and in addition, the sound wave that produces the cavitation induces an acoustic-optic effect [24]. Also, the presence of collapsing bubbles can be inferred from second harmonic generation [29]. With laser scattering method, most of the temporal and spatial scales related to the dynamics of a cavitation bubble can be captured; however this method is not able to give qualitative information about bubbles or non-spherical bubbles. In addition, the theory behind this method is only applied to spherical shape bubbles considering that all forms of bubbles, spherical and non-spherical, are produced in clinical application of ultrasound [30]. In this technique the volume of the sample is very small and unrestricted visual access at high magnification is required [30]. The acoustic detection technique has the positive aspect of in vivo application, however the size of the initial cavitation bubble and the amplitude of the ultrasound that produced the cavitation bubbles, affect the timing and amplitude of the cavitation bubbles’ emissions.

Analyzer-based X-ray imaging (ABI) [31] has the potential to detect and visualize ultrasound cavitation bubbles in thick, optically opaque materials such as in vivo tissue relying on the high penetration of X-rays and a sensitivity to small angle refraction. As such, ABI can visualize and characterize properties of the cavitation bubbles in vivo without having any influence on the bubbles or vice versa.

The present authors have used ABI for visualizing cavitation bubbles from a high intensity sonochemistry system [32]. In that work, the operating conditions are well beyond that used for any physical therapy or imaging application (130 W and 20 kHz). The system relies on generating cavitation bubbles for cell disruption. The present paper addresses cavitation bubble formation in a type of ultrasound system commonly used for physical therapy applications (14 W and 0.88 MHz) where one might not expect to observe cavitation bubbles. Again, the use of an X-ray method can allow observation of cavitation in opaque systems without interacting with the cavitation process.

### X-ray ABI

X-ray ABI is a phase sensitive imaging technique that can detect subtle projected density and thickness variations in materials such as tissue. As a collimated X-ray beam travels through the object being imaged, it may be refracted, scattered or absorbed. Small structures such as a bubble in tissue or water will refract the X-rays through very small angles. With ABI, these small angles can create contrast based on the very narrow reflectivity curve of the analyzer crystal placed after the object. Thus the ABI technique is particularly well suited to visualize interfaces between features within soft tissues such as bubbles. This leads to very high contrast for some tissues such as lung particularly when the analyzer is placed at the peak position. The alveoli appear as a “bubbly” structure which very effectively refracts the X-rays and thus create contrast. The effect of multiple refraction events by several alveolar interfaces creates a scatter distribution (ultra-small angle X-ray scattering) of the X-rays which effectively removes X-rays from their original collimated trajectory. This scatter distribution can be very effectively interrogated by the analyzer crystal.

**Fig. 1:**
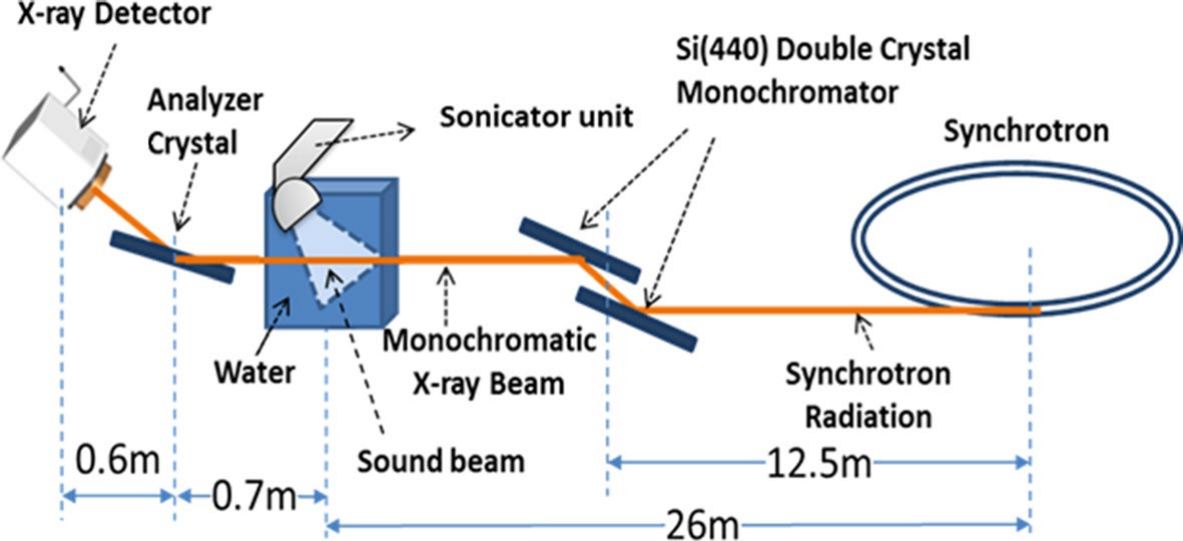
Schematic of the ABI set up performed for imaging therapeutic ultrasound induced cavitation bubbles in water at Canadian Light Source

Detecting ultrasound cavitation bubbles in tissues can be simplified by ABI. The bubbles will be of a transient nature and should provide enough contrast to be imaged in a time averaged exposure. For example, a single air bubble the same size as a detector pixel can generate ~20 % contrast compared to a region not containing a bubble. Applying ABI the density of stationary and moving bubbles in the tissue and intravascular can be indirectly inferred by measuring the ultra-small angle X-ray scattering distribution in the zone of images where bubbles are formed. However, at the top or peak spot of the analyzer, there is a recognizable loss of intensity due to scattering from the bubbles. With ABI, the X-ray imaging beam is prepared, or collimated, by Bragg diffraction from a perfect crystal monochromator which is typically made with silicon crystals (see Fig. 1). A double crystal arrangement is applied so that the imaging energy can be varied while the exit beam is in the same direction as the incident synchrotron beam. The imaging energy is usually selected based on the sample’s composition, thickness and features of interest. The object is located in the beam with an analyzer crystal downstream of the object before the detector. The analyzer is parallel to the double monochromator crystals and is of the same direction, reflection and crystal type. In this arrangement, as the analyzer is locked in angle near the Bragg angle for the energy and lattice plans selected, the intensity profile is called a rocking curve. When the X-ray beam passes through the sample being imaged, the X-rays are refracted at the interfaces of features or structures in the sample through angles of a few nanoradians to microradians. The analyzer can be adjusted over these angular ranges and the character of the image is greatly affected by the angular setting. The analyzer at the peak setting is sensitive to X-rays redirected in angle such as scatter and removes it from the image. The ABI experimental procedure is explained in more detail in other publications [31] [33, 34]. ABI has demonstrated a noticeable ability to image structures that have interfaces between materials of various density such as that between air and water in a bubble. The cavitation bubble will be of a transient nature; however, they are continuously created and may provide enough contrast to be imaged in a time averaged exposure.

## Methods

### Materials

Tap water was used as the sample to produce ultrasound induced cavitation bubbles in it. The tap water was pre-boiled and let stand for 48 h in advance the experiment.

### Ultrasound treatment

**Fig. 2:**
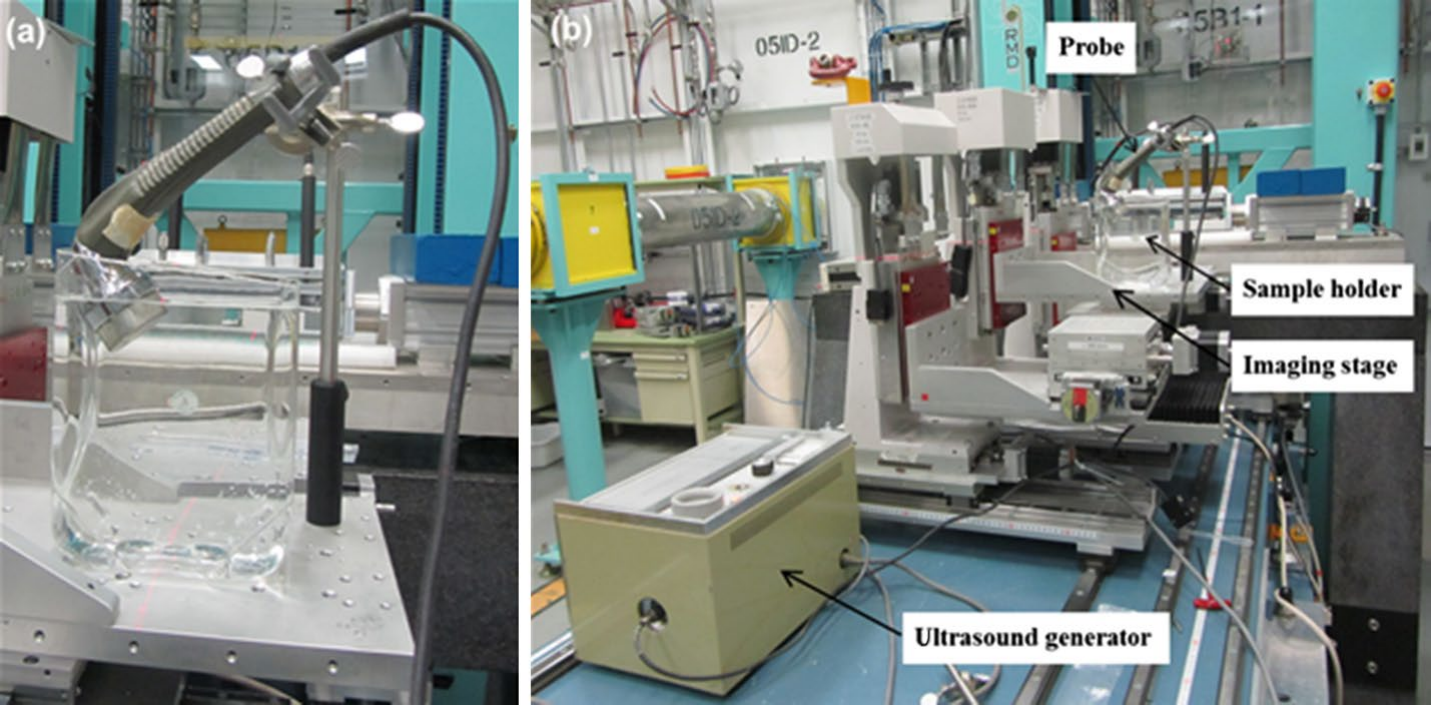
Preparation of the sample for therapeutic ultrasound treatment and X-ray imaging: **a** mounting the ultrasound probe on the scanning stage and inserting probe inside and at 45° angle of sample/water; **b** setting the signal generator/amplifier on the table and setting the experimental system

Sonication of water was performed by means of a therapeutic ultrasound device (Burdick ultrasound therapy unit model UT-420A), with specifications of 0.8835 MHz, 3.5 W/cm^2^, 14 W, connecting a probe with size of 13 cm^2^. The ultrasound therapy unit was brought to the Biomedical Imaging and Therapy (BMIT) bend magnet beamline (BM 05B1-1) of the Canadian Light Source (CLS), Saskatoon, SK, Canada. A tissue culture flask (300 cm^2^, 1900 ml) was used as sample holder for this experiment. The top part of the flask was cut off by a foam cutter hot knife. Then the sample holder was placed on the imaging stage in such a way that the largest surface of the flask faces the imaging beam. The probe was mounted on the specimen stage at the experimental end station of BMIT beamline using a support stand. The sample holder was filled with pre-boiled tap water (to a height of 100 mm) and the probe was placed in the water top at 2.7 cm below the surface of the water with a 60° angle (Fig. 2a). The probe was connected to the signal generator/amplifier that was placed on the table away from the X-ray beam (Fig. 2b). The pulse mode was adjusted for continuous acoustic irradiation and the power output of the processor was adjusted at the maximum output. An electronic switch that was connected to the ultrasonic processor foot switch interface was developed and controlled through National Instrument data acquisition and control system. This arrangement allowed the control of the ultrasonic processor through LabVIEW software (National Instruments Corp., Austin, TX, USA), and collect long image sequences automatically with the sonicator turned on and off as needed in the different parts of the experimental sequence. Two visual monitor cameras were adjusted at the sample and at the generator in order to ensure the synchronize operation of the probe and generator along with the Lab VIEW software during image sequence collection. The best mounting position and spatial orientation of the ultrasound and sample holder with respect to the incident beam was identified so that the X-ray beam horizontally covered the entire width of the sample holder for imaging. Images were taken at four different positions. The experimental system position for various fields of views at each experimental condition was changed by adjusting the scanning stage.

### Synchrotron imaging

Imaging of therapeutic ultrasound induced cavitation bubbles was performed at the CLS synchrotron source (BMIT-BM 05B1-1). A highly collimated, monochromatic, X-ray beam with maximum horizontal beam size of 250 mm and maximum vertical beam size of 8.0 mm produced by a bend magnet (1.354 T) was used for imaging. The X-ray beam with photon energy of 40 keV was prepared by the double crystal monochromator reflection of Si (4,4,0) to provide high contrast images. Depending on the chosen photon energy, the X-ray beam with vertical beam size of 4.0 mm and horizontal beam size of 250 mm at the sample location and the detector was applied for imaging experiments. Images were collected by an X-ray camera (VHR-90, Photonic Science, Mountfield, East Sussex, UK) with gadolinium oxysulphide scintillator layer having a projected density of 7.5 mg/cm^2^ and area of 74.9 mm × 49.9 mm (4008 × 2672 pixels) with an effective pixel size of 18.5 μm. Pixel binning of 4 × 4 was applied (optical pixel size of 74 μm × 74 μm) and the region of interest of 100 × 77 pixels (7.4 × 5.7 mm) was selected. Planar ABI was performed for imaging of therapeutic ultrasound induced cavitation bubbles in this study. A schematic of the ABI system applied is shown in Fig. 2. The distance between the sample and the X-ray source was about 26 m and the distance between the double crystal monochromator and sample was approximately 13.5 m. The monochromator—analyzer used in ABI was a silicon (4,4,0) configuration. The analyzer was adjusted very close to the top of the rocking curve. The distance between the analyzer crystal and detector was 0.6 m, and the distance between the sample and the analyzer was 0.7 m as demonstrated in the Fig. 1.

*Multiple image contrast* technique was used for collection of images at each distance from the tip of the probe. In this technique a large number of images in sequence mode were collected to improve the signal to noise ratio and to minimize the effects of the small drift of the analyzer crystal. The imaging sequence contained 7000 on–off cycles. In each cycle 2 images were captured. First an image was collected when the ultrasound was turned on, immediately after that the ultrasound was turned off and after a 500 ms delay another image was collected. The time necessary to complete one cycle is small and both images were collected practically at same point of the analyzer rocking curve. More details on this method of imaging can be found of the previous work of authors [32]. When the ultrasound was on, the output power of sonicator was set for 14 W. The sample area of 17.75–31.75 mm below the lowest point of the tip of the probe in the sample was imaged. The sample over 14 mm range was scanned by taking 4 frames and incrementing the position of the scanning stage by 3.5 mm between each frame (at four different locations of 19.5, 23, 26.5, and 30 mm below the tip of the probe). The exposure time for each frame was 2.5 s, selected based on the intensity of the X-ray beam. In total 7000 images with ultrasound on and 7000 images with ultrasound off were acquired. Then the two resulting summed image sets were divided by each other. The initial results were calculated and analyzed by ImageJ software program. Then, a dedicated program was written in Interactive data language (IDL) software program (Exelis Visual Information Solution, Inc., Boulder, Colorado, USA) and the final results were analyzed. The intensity ratio (on divided by off) was evaluated for each image set. At 40 keV the total radiation exposure was about 17 Gy for the 14,000 images.

## Result and discussion

### Preliminary experiments

**Fig. 3:**
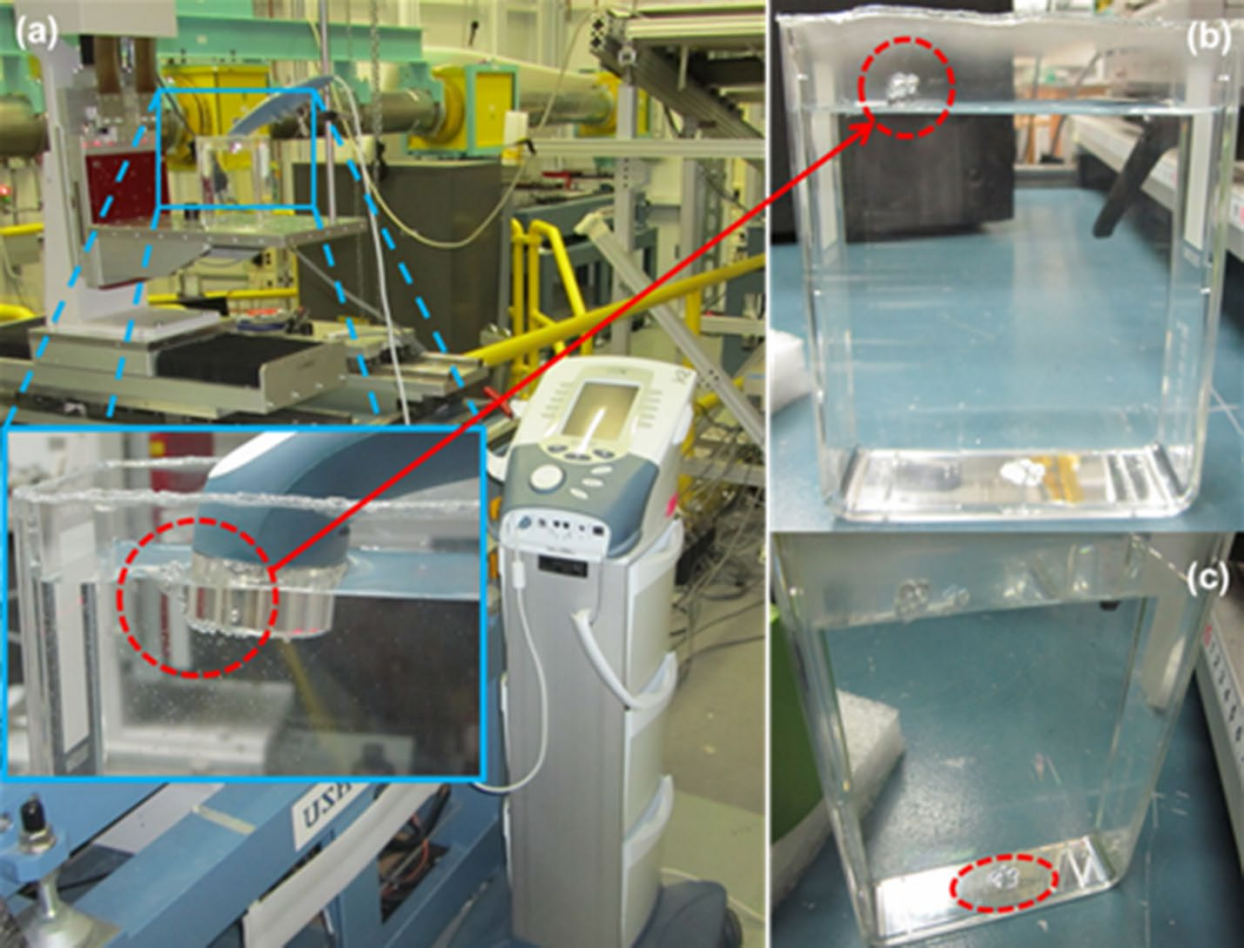
The experimental set up of the preliminary experiments and the observation of the sample container melting (**a**) the melted sign of container on the wall (**b**) and at the center bottom of the container (**c**)

Preliminary experiments were performed by means of a therapeutic ultrasound system (Intelect advanced, Chattanooga group, a division of encore medical, L.P. 2005) with the specification of 0.8835 MHz, Duty cycle 100 %, 1 W/cm^2^, 4 Watt, Duty frequency 100 Hz, treatment time of 60 min, and probe size of 5 cm^2^. During preliminary experiments it was observed that at two specific points of the sample holder, at the wall close to the probe (Fig. 3b), and also at the bottom center of sample holder (Fig. 3c), the sample holder was melting. This means that the transducer field was consistently energetically non-uniform with some spatial ‘hot-spots’ occurring during the application which lead to burning points (Fig. 3a). These non-uniformities in the acoustic field, simultaneously with the high time-averaged intensities produced, can cause patient burns (Advisory group on non-ionising radiation 2010). This issue has become the issuing of a recent safety in Scotland [35].

### Main experiments

**Fig. 4:**
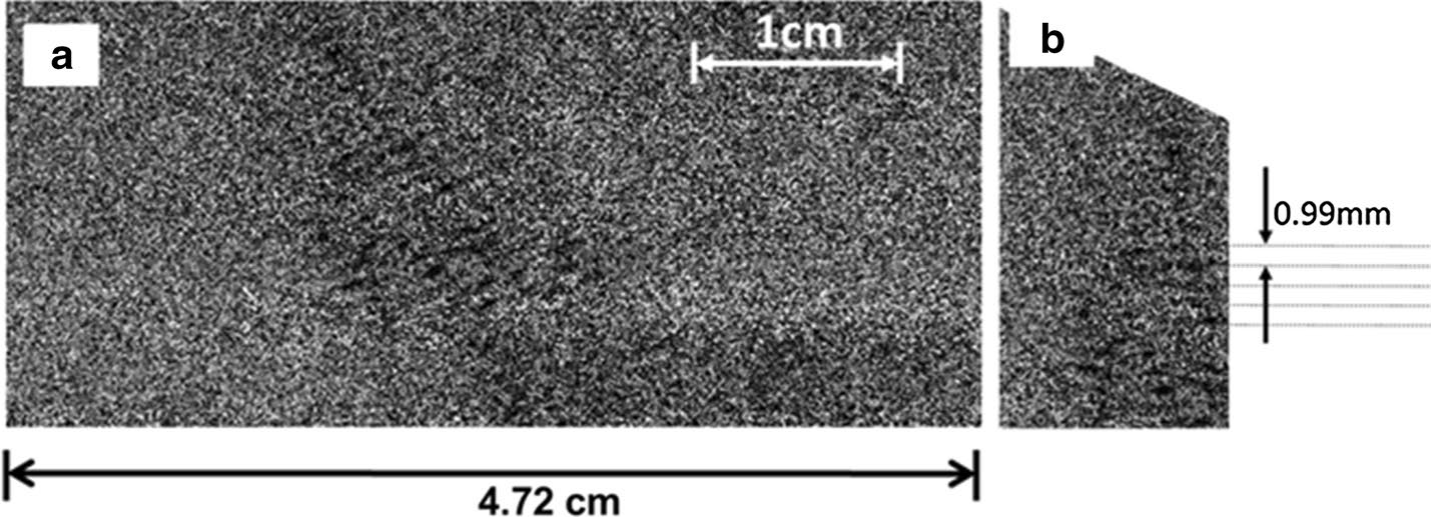
*Vertical* scan of the whole ultrasound beam of a therapeutic ultrasound system below the tip of the probe at 0.8835 MHz and 14 W (**a**). The sequence form of cavitation bubbles’ location with approximately same intervals between sequences (**b**)

Ultrasound induced cavitation bubbles produced by the therapeutic system (physical therapy device with unfocused beam) were detected during multiple image ABI experiments. The processed results from the 7000 on–off images, showed the region of cavitation bubbles in the area below the probe, with 60 degree angle from the water surface along the direction of ultrasound probe. The scan of the ultrasound beam in the area between 17.75 and 31.75 mm below the surface of the probe is shown in Fig. 4a. The presence of cavitation bubbles was detected as loss of intensity that appears as the dark shadow in the area below the ultrasound probe, with a 60° angle at the center of the image (Fig. 4a). The spatial structure of the ultrasonic field can determine the most probable location of the cavitation bubbles. The spatial structure of the beam can be controlled by factors such as the shape and dimension of the source with respect to the wavelength of the ultrasound propagated in the sample, and also the pulsed or continuous mode of sound propagation in sample. In this study the shape of the probe was a circularly symmetric source and the ultrasound was propagated in the sample in a continuous mode at a single frequency and amplitude. The analysis of such ultrasound beams has been well studied in the literature [36, 37]. As the speed of sound in water is around 1480 ms^−1^, at frequency of 0.8835 MHz, the wavelength in water and soft tissue is approximately 1.675 mm in which the wavelength was calculated as follow:

1 $$\uplambda = {\text{c}}/{\text{f}}$$

where λ is the wavelength in mm, c is the velocity of sound in mm/s, and f is the frequency in Hz. Based on a previous study performed by the authors, the current multiple imaging technique was established for visualization of ultrasound induced cavitation bubbles [32]. The signal to noise ratio from a single ultrasound—induced cavitation bubble is very weak. The features of cavitation bubbles in water are not revealed by a single ABI image and the signal to noise ratio is improved by taking more images and summing them. The number of photons in the final, averaged image is raised by increasing the number of images and as a result more features of bubbles are revealed due to the increased signal-to-noise.

**Fig. 5:**
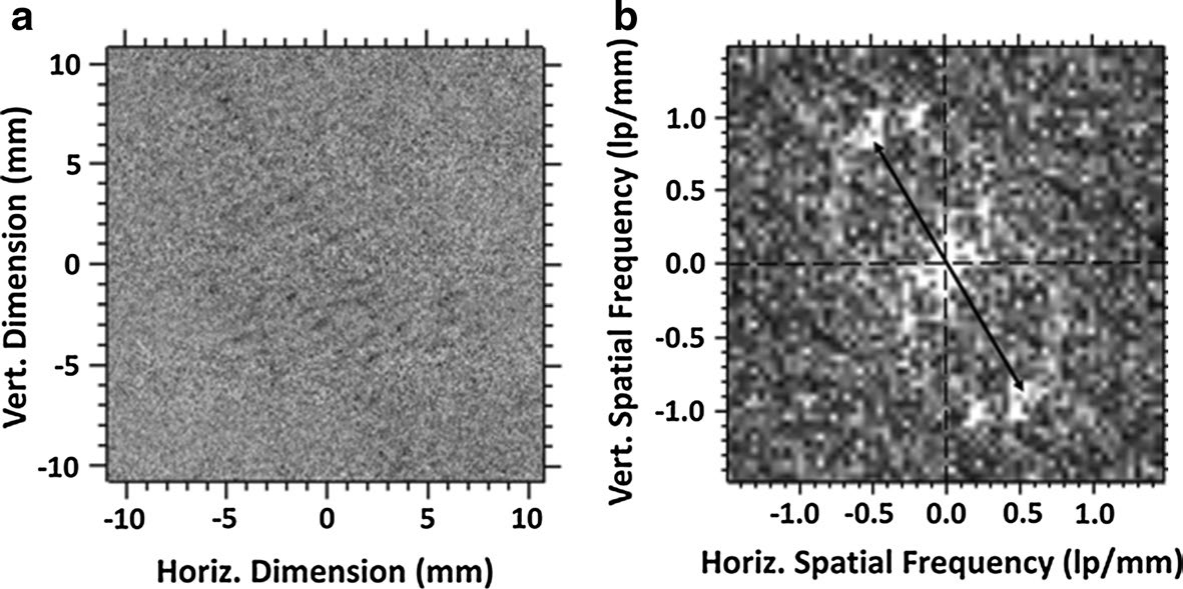
The region selected from Fig. 4a for Fourier analysis **a** The power spectrum obtained from Fig. 4a. The *black line* shows the wave vector associated with the strongest Fourier component whose magnitude is approximately 1.15 line-pairs/mm (**b**)

As shown in Fig. 4b, it was observed that the cavitation bubbles appeared in a periodic pattern. The most likely reason for this spatial structure can be the production of a standing wave in the sample. In case the pattern in Fig. 4 is the formation of an acoustic standing wave field due to trapped cavitation induced bubbles, then the standing wave patterns should be one-half of the wave length. To investigate this, the distance between the two consecutive parallel lines of the pattern in Fig. 4b was measured as 0.99 mm. The periodicity can also be obtained from Fourier analysis (Fig. 5b) and was found to be 1.15 line-pairs (lp) per mm. Figure 5b is a spatial power spectrum of the acoustic field region shown in Fig. 5a and the bright regions indicate spatial periodicities of higher amplitude. Note the somewhat dual circular appearance of the power spectrum which indicates there are multiple directions which describe the acoustic pattern. We have selected the component that corresponds to the pattern most prominent in Figs. 4b, 5a. This is the reciprocal of the periodicity distance and leads to a spatial periodicity of 0.870 mm. Therefore, the distance between the two consecutive parallel lines of the pattern is 0.99 mm from Fig. 4b and the Fourier analysis giving 0.870 mm is close to the theoretically calculated value of 0.838 mm meaning that the assumption of having acoustic standing wave and also the first harmonic can be correct. However, it should be noted that the geometry of the container did not support the formation of the standing wave completely; there was no surface perpendicular to the acoustic wave (or surface parallel to the probe surface). The pattern of Fig. 4 indicates that an acoustic standing wave was formed in the container at applied therapeutic ultrasound frequency, and the generated cavitation bubbles were trapped at the nodes or anti-nodes of the acoustic standing wave field, and form the temporary stationary bubbles. The fact that the container surfaces were not parallel to the probe surface may give rise to a complex standing wave pattern as indicated by the Fourier power spectrum (Fig. 5b) where there appear to be several standing waves which in that figure which form a somewhat circular pattern. The stationary bubbles dance/vibrate at their zone and act as sound scatterers [38]. These bubbles finally collapse as the ultrasound goes through off cycle. Although, the vibration of bubbles causes the scattered acoustic energy to modulate the exciting carrier signal, the constant generation of these bubbles at same locations and their collapse afterward can release energy and possibly cause damage.

**Fig. 6:**
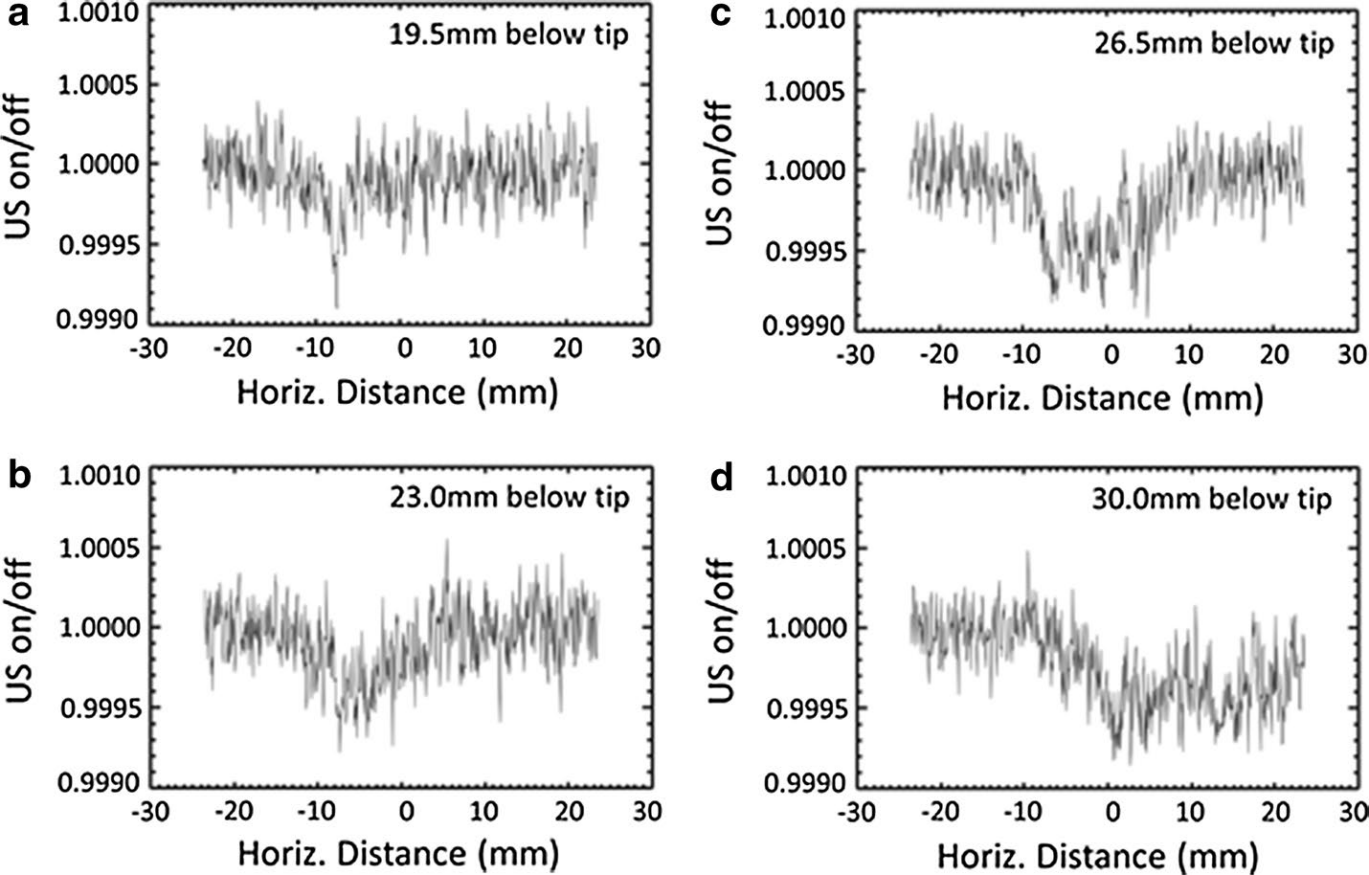
The intensity profile of images taken of the cavitation bubbles at different distances from the tip of the probe; 19.5 mm (**a**), 23 mm (**b**), 26.5 mm (**c**), 30 mm (**d**)

The line intensity profile of images at different imaging distances from the tip of the probe is shown in Fig. 6. The line intensity profile of each image obtained from the average of 31 image lines about the center of each image. The intensity profiles demonstrate that the intensity loss as a result of the presence of bubbles. This loss has the highest magnitude at the closest distance to the probe (19.5 mm below tip) and weakens with distance from the probe tip. This demonstrates that the density of cavitation bubbles is higher close to the probe and declines with distance away from the probe. In addition the width of the dip in the graph rises with distance from the tip of the probe, showing the horizontal area in which cavitation bubbles are present. As shown in Fig. 6, the width of the beam is much smaller at 19.5 mm (Fig. 6a) than 30 mm (Fig. 6d) away from the tip of the probe. It is observed from Fig. 5 that the acoustic beam can be cone shaped meaning that the width of distribution of cavitation bubbles increases by increasing the distance from the probe. The width of the intensity dips in the line plots across the image in Fig. 6 confirms the rise in width of cavitation bubbles’ distribution in the sample with distance from the probe.

## Conclusion

The pattern of cavitation bubbles in water driven by a 0.8835 MHz therapeutic ultrasound system at 14 watt output power were detected by synchrotron X-ray ABI. Since the flux of X-ray at 40 keV on the BMIT beamline is quite low, the imaging time was long. Although the acquisition time was somewhat long, the pattern of induced cavitation bubbles was revealed. The cavitation bubbles’ pattern was observed in repetitive lines. The calculated distance between intervals revealed that the distance of frequent cavitation lines (intervals) is one-half of the acoustic wave length consistent with standing waves. The presence of bubbles was observed as a time-averaged bubble region. The density and location of bubbles were inferred indirectly by measuring the ultra-small angle X-ray scattering distribution in the region of images where bubbles (cavitation) were formed. This set of experiments demonstrates the utility of synchrotron ABI for visualizing cavitation bubbles formed in water by clinical ultrasound systems working at high frequency and output powers as low as a therapeutic system. This can be the first step toward more detailed characterization of cavitation bubbles formation in other clinical acoustic systems such as HIFU and lithotripsy.

## References

[1] Chemat S, Lagha A, AitAmar H, Bartels PV, Chemat F (2004). Comparison of conventional and ultrasound-assisted extraction of carvone and limonene from caraway seeds. Flavour Fragr J.

[2] Ikeda T, Yoshizawa S, Tosaki M, Allen JS, Takagi S, Ohta N (2006). Cloud cavitation control for lithotripsy using high intensity focused ultrasound. Ultrasound Med Biol.

[3] Delius M, Mueller W, Goetz A, Liebich H, Brendel W (1990). Biological effects of shock waves: kidney hemorrhage in dogs at a fast shock wave administration rate of fifteen Hertz. J Lithotripsy Stone Dis..

[4] Evan AP, Willis LR, McAteer JA, Bailey MR, Connors BA, Shao Y (2002). Kidney damage and renal functional changes are minimized by waveform control that suppresses cavitation in shock wave lithotripsy. J Urol..

[5] Zhu S, Dreyer T, Liebler M, Riedlinger R, Preminger GM, Zhong P (2004). Reduction of tissue injury in shock-wave lithotripsy by using an acoustic diode. Ultrasound Med Biol.

[6] Zhou Y-F (2011). High intensity focused ultrasound in clinical tumor ablation. World J Clin Oncol.

[7] Suslick K (1994). The yearbook of science and the future.

[8] Daniels S, Kodama T, Price D (1995). Damage to red blood cells induced by acoustic cavitation. Ultrasound Med Biol.

[9] Child SZ, Hartman CL, Schery LA, Carstensen EL (1990). Lung damage from exposure to pulsed ultrasound. Ultrasound Med Biol.

[10] Holland CK, Deng CX, Apfel RE, Alderman JL, Fernandez LA, Taylor KJ (1996). Direct evidence of cavitation in vivo from diagnostic ultrasound. Ultrasound Med Biol.

[11] O’Brien WD, Zachary JF (1994). Comparison of mouse and rabbit lung damage exposure to 30 kHz ultrasound. Ultrasound Med Biol.

[12] O’Brien WD, Zachary JF (1996). Rabbit and pig lung damage comparison from exposure to continuous wave 30-kHz ultrasound. Ultrasound Med Biol.

[13] O’Brien WD, Zachary JF (1997). Lung damage assessment from exposure to pulsed-wave ultrasound in the rabbit, mouse, and pig. Ultrason Ferroelectr Freq Control IEEE Trans.

[14] O’Brien WD, Simpson DG, Frizzell LA, Zachary JF (2001). Superthreshold behavior and threshold estimates of ultrasound-induced lung hemorrhage in adult rats: role of beamwidth. IEEE Trans Ultrason Ferroelectr Freq Control.

[15] O’Brien WD, Kramer JM, Waldrop TG, Frizzell LA, Miller RJ, Blue JP (2002). Ultrasound-induced lung hemorrhage: role of acoustic boundary conditions at the pleural surface. J Acoust Soc Am..

[16] O’Brien WD, Simpson DG, Ho M-H, Miller RJ, Frizzell L, Zachary JF (2003). Superthreshold behavior and threshold estimation of ultrasound-induced lung hemorrhage in pigs: role of age dependency. Ultrason Ferroelectr Freq Control IEEE Trans.

[17] Tarantal AF, Canfield DR (1994). Ultrasound-induced lung hemorrhage in the monkey. Ultrasound Med Biol.

[18] ter Haar G (2007). Therapeutic applications of ultrasound. Prog Biophys Mol Biol.

[19] Sokka S, King R, Hynynen K (2003). MRI-guided gas bubble enhanced ultrasound heating in in vivo rabbit thigh. Phys Med Biol.

[20] Vykhodtseva N, Hynynen K, Damianou C (1995). Histologic effects of high intensity pulsed ultrasound exposure with subharmonic emission in rabbit brain in vivo. Ultrasound Med Biol.

[21] Husseini GA, de la Rosa MAD, Richardson ES, Christensen DA, Pitt WG (2005). The role of cavitation in acoustically activated drug delivery. J Controlled Release.

[22] Coussios C, Farny C, Ter Haar G, Roy R (2007). Role of acoustic cavitation in the delivery and monitoring of cancer treatment by high-intensity focused ultrasound (HIFU). Int J Hyperth.

[23] Lizzi F, Coleman D, Driller J, Silverman R, Lucas B, Rosado A, editors. A therapeutic ultrasound system incorporating real-time ultrasonic scanning. IEEE 1986 Ultrasonics Symposium. 1986, IEEE.

[24] Frohly J, Labouret S, Bruneel C, Looten-Baquet I, Torguet R (2000). Ultrasonic cavitation monitoring by acoustic noise power measurement. J Acoust Soc Am..

[25] Philipp A, Delius M, Scheffczyk C, Vogel A, Lauterborn W (1993). Interaction of lithotripter-generated shock waves with air bubbles. J Acoust Soc Am..

[26] Sass W, Matura E, Dreyer H, Folberth W, Seifert J (1993). Lithotripsy-mechanisms of the fragmentation process with focussed shock waves. Electromedica..

[27] Zhong P, Cioanta I, Cocks FH, Preminger GM (1997). Inertial cavitation and associated acoustic emission produced during electrohydraulic shock wave lithotripsy. J Acoust Soc Am..

[28] Pishchalnikov YA, Sapozhnikov OA, Bailey MR, Williams JC, Cleveland RO, Colonius T (2003). Cavitation bubble cluster activity in the breakage of kidney stones by lithotripter shockwaves. J Endourol.

[29] Yoshizawa S, Yasuda J, Umemura S (2013). High-speed observation of bubble cloud generation near a rigid wall by second-harmonic superimposed ultrasound. J Acoust Soc Am.

[30] Cleveland RO, McAteer JA (2007). The physics of shock wave lithotripsy. Smith’s Textb Endourol.

[31] Chapman D, Thomlinson W, Johnston R, Washburn D, Pisano E, Gmür N (1997). Diffraction enhanced X-ray imaging. Phys Med Biol.

[32] Izadifar Z, Belev G, Izadifar M, Izadifar Z, Chapman D (2014). Visualization of ultrasound induced cavitation bubbles using the synchrotron X-ray analyzer based imaging technique. Phys Med Biol.

[33] Kelly ME, Beavis RC, Fourney DR, Schültke E (2006). Diffraction-enhanced imaging of the rat spine. Can Assoc Radiol J.

[34] Zhong Z, Thomlinson W, Chapman D, Sayers D (2000). Implementation of diffraction-enhanced imaging experiments: at the NSLS and APS. Nucl Instrum Methods Phys Res Sect A.

[35] Safety Action Notice SAN (SC) 06/44. Physiotherapy ultrasound machines: calibration of acoustic power/intensity. Edinburgh: Scottish Healthcare Supplies. 2006.

[36] Duck FA, Baker AC, Starritt HC (1998). Ultrasound in medicine.

[37] Kinsler LE, Frey AR, Coppens AB, Sanders JV. Fundamentals of acoustics. In: Lawrence E Kinsler, Austin R Frey, Alan B Coppens, James V Sanders, editors. Fundamentals of acoustics, 4th edn. pp 560 ISBN 0-471-84789-5 Wiley-VCH, December 1999. 1999. pp. 1.

[38] Scherf WW. Amplitude modulation of a stationary acoustic field by cavitation bubbles: DTIC Document. 1971.

